# Assessing biotic and abiotic effects on forest productivity in three temperate forests

**DOI:** 10.1002/ece3.6516

**Published:** 2020-06-30

**Authors:** Qingmin Yue, Minhui Hao, Xiaoyu Li, Chunyu Zhang, Klaus von Gadow, Xiuhai Zhao

**Affiliations:** ^1^ Research Center of Forest Management Engineering of State Forestry and Grassland Administration Beijing Forestry University Beijing China; ^2^ Faculty of Forestry and Forest Ecology Georg‐August‐University Göttingen Germany; ^3^ Faculty of AgriSciences Stellenbosch University Matieland South Africa

**Keywords:** biodiversity and productivity relationships, biotic effects, forest productivity, species diversity, stand density, structural diversity

## Abstract

1. It is well understood that biotic and abiotic variables influence forest productivity. However, in regard to temperate forests, the relative contributions of the aforementioned drivers to biomass demographic processes (i.e., the growth rates of the survivors and recruits) have not received a great deal of attention. Thus, this study focused on the identification of the relative influencing effects of biotic and abiotic variables in the demographic biomass processes of temperate forests.
2. This study was conducted in the Changbai Mountain Nature Reserve, in northeastern China. Based on the observational data collected from three 5.2‐hectare forest plots, the annual above‐ground biomass (AGB) increment (productivity) of the surviving trees, recruits, and the total tree community (survivors + recruits) were estimated. Then, the changes in the forest productivity in response to biotic variables (including species diversity, structural diversity, and density variables) along with abiotic variables (including topographic and soil variables) were evaluated using linear mixed‐effect models.
3. This study determined that the biotic variables regulated the variabilities in productivity. Density variables were the most critical drivers of the annual AGB increments of the surviving trees and total tree community. Structural diversity enhanced the annual AGB increments of the recruits, but diminished the annual AGB increments of the surviving trees and the total tree community. Species diversity and abiotic variables did not have impacts on the productivity in the examined forest plots.
4. The results highlighted the important roles of forest density and structural diversity in the biomass demographic processes of temperate forests. The surviving and recruit trees were found to respond differently to the biotic variables, which suggested that the asymmetric competition had shaped the productivity dynamics in forests. Therefore, the findings emphasized the need to consider the demographic processes of forest productivity to better understand the functions of forests.

## Graphical abstract

Density variables were the most critical drivers of the AGB increments of the surviving trees. Structural diversity enhanced the AGB increments of the recruits, but diminished the annual AGB increments of the surviving trees. Species diversity and abiotic variables did not have impacts on the productivity in the examined forest plots.

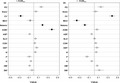

## 1 INTRODUCTION

It has been determined that forests hold an estimated 85% of the global terrestrial biomass and 60% of gross primary productivity. Therefore, forest ecosystems play important roles in regulating global carbon cycles (Houghton, Hall, & Goetz, 2009; Randolph, Green, Belmont, Burcsu, & Welch, 2005). However, with the unprecedented biodiversity loss rates throughout the world, there has been increasing concern that species loss may affect the functioning of forest ecosystems (Cardinale et al., 2012; Hooper et al., 2012). The majority of the observational evidence has indicated that there are positive relationships between biodiversity and ecosystem functioning (Duffy, Godwin, & Cardinale, 2017; Vila, Vayreda, Gracia, & Ibanez, 2003; Zhang, Chen, & Reich, 2012). Furthermore, it is now assumed that strong abiotic forces and complex interactions obscure biodiversity effects (Ali et al., 2019a; Duffy et al., 2017; Ratcliffe et al., 2016; van der Sande et al., 2018). Different demographic processes of biomass dynamics (e.g., biomass increments of the surviving and recruit trees) may respond differently to the biotic and abiotic variables (de Avila et al., 2018; Finegan et al., 2015). However, the determination of the relative importance of the aforementioned biotic and abiotic effects on the demographic processes has received little attention, despite its importance for understanding biomass dynamics and the relationships between biodiversity and ecosystem functioning (de Avila et al., 2018; van der Sande et al., 2017; Yuan et al., 2019).

It is commonly believed that species richness (hereafter referred to as SR) is a representative metric of biodiversity (Letcher & Chazdon, 2009; Ruiz‐Benito et al., 2014). In addition, it is considered that phylogenetic diversity (hereafter referred to as PD) reflects the evolutionary history among species and displays a strong correlation with productivity (Cadotte, Cardinale, & Oakley, 2008; Flynn, Mirotchnick, Jain, Palmer, & Naeem, 2011). The majority of the previous related studies indicated that positive diversity effects were detected in forests with enhanced productivity levels (Duffy et al., 2017; Liang et al., 2016; Luo, Liang, Cazzolla Gatti, Zhao, & Zhang, 2019; Zhang et al., 2012). However, negative, nonsignificant, and unimodal relationships were also found in natural forests (Tobner et al., 2016; Vila et al., 2003; Zhang et al., 2012). Two main mechanisms may potentially explain the aforementioned positive relationships: niche complementarity and selection effects (Loreau & Hector, 2001; Ruiz‐Benito et al., 2014). The niche complementarity effect mechanism is generally based on the assumption that combination of different species will facilitate the use of limited resources, thereby promoting productivity (Cadotte, 2017; Tilman, Lehman, & Thomson, 1997). Meanwhile, the selection effect mechanism proposes that high diversity increases the probability of including the most productive species (Loreau & Hector, 2001).

In addition to species diversity, structural diversity (e.g., reflecting the horizontal and vertical heterogeneity of a stand) also tends to promote productivity through the effective utilization of resources through niche complementarity and facilitation processes (Fotis et al., 2018; Ouyang et al., 2019; Pach & Podlaski, 2015; Zhang & Chen, 2015). However, recent studies have also reported that structural diversity may have negative or nonsignificant effects on productivity due to the asymmetric competition for light, competitive exclusion, and selection effects (Ali, 2019; Bourdier et al., 2016; Forrester & Bauhus, 2016; Kunz et al., 2019; Zhang et al., 2017). Additionally, stand density (e.g., initial forest biomass or the number of stand stems) may exert considerable influence on forest productivity (Corral‐Rivas et al., 2016; Lohbeck, Poorter, Martinez‐Ramos, & Bongers, 2015; Ouyang et al., 2019). On the one hand, stand density can increase productivity by enhancing canopy packing and light interception (Forrester & Bauhus, 2016). On the other hand, due to the negative effects of density, the number of neighborhood trees may have negative effects on the performances of the focus trees at a neighborhood scale (Chen et al., 2016; Fortunel et al., 2018). Therefore, the direct effects of biotic variables (e.g., species diversity, structure diversity, and density) on driving productivity at the local level remain a controversial topic.

Abiotic variables tend to be steady drivers of productivity, since they are known to determine resource availability for plant growth and survival (van der Sande et al., 2017; Yuan et al., 2018). For example, poor soil conditions were found to have strongly limited forest productivity in the Guyanese Forest (van der Sande et al., 2018), while good soil conditions were determined to promote subtropical forest growth (Ouyang et al., 2019). Furthermore, at the stand level, differences in such topographic variables as elevation, slope, aspect, and convexity may create micro‐environment heterogeneity in resource inequality (light, water, and soil fertility), subsequently negatively affecting forest productivity (Fortunel et al., 2018; van der Sande et al., 2018). In previous studies, Hao, Zhang, Zhao, and von Gadow (2018) revealed the fundamental roles of topographic conditions in determining forest stand volumes, biomass, species diversity, and productivity levels in temperate forests.

In the present study, the goal was to quantify the effects of biotic variables (species diversity, structural diversity, and stand density) and abiotic conditions (soil and topographic conditions) on the demographic processes of forest productivity (e.g., the growth rates of the surviving and recruit trees) in three 5.2‐hectare field plots in northeastern China. Specifically, the following questions were addressed in this study:

1. How do biotic and abiotic variables affect productivity in the three examined temperate forests?
2. What is the relative importance of the biotic and abiotic variables in relation to the demographic processes of forest productivity?

## 2 METHODS

### 2.1 Study site and sampling set

**FIGURE 1:**
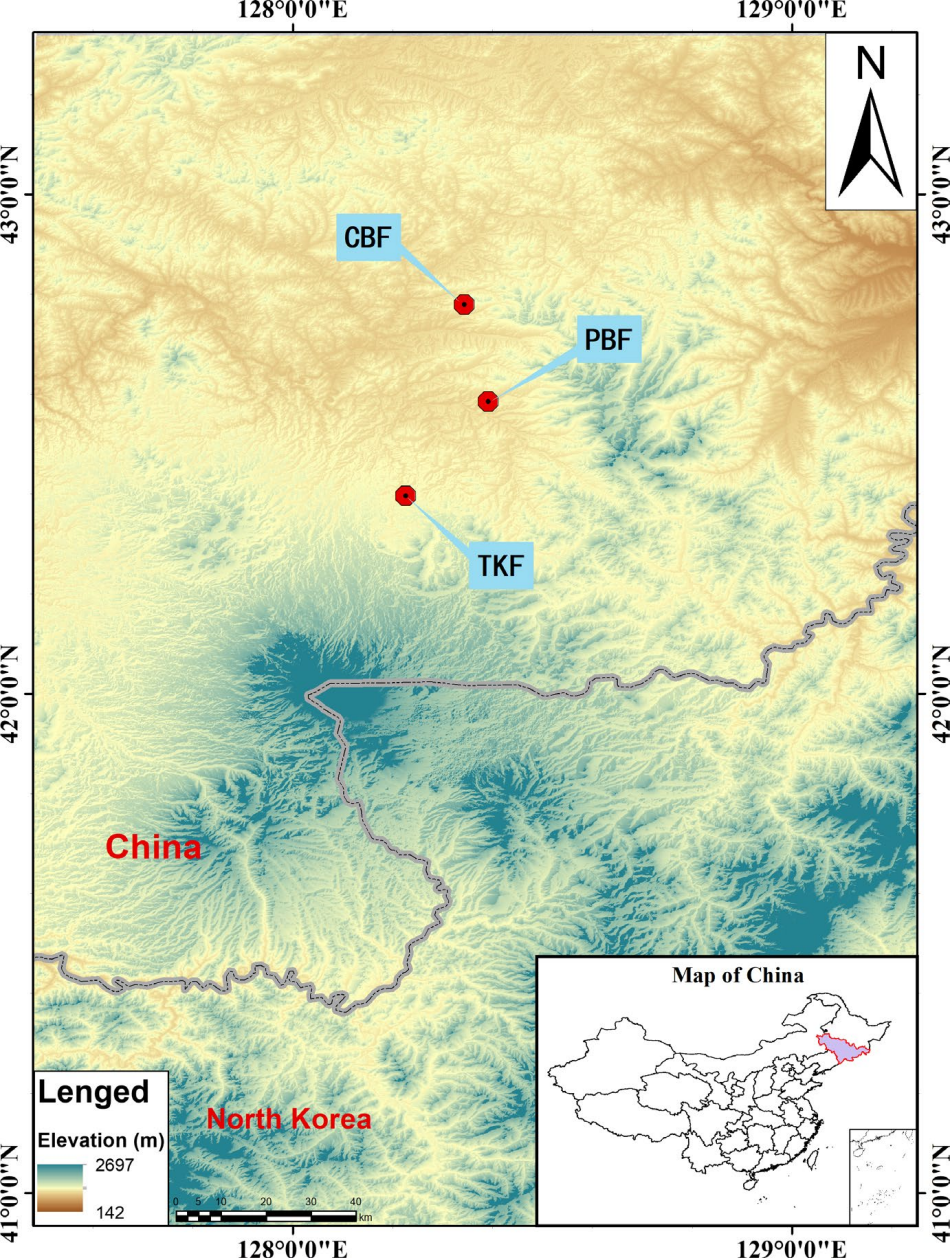
Location map of the study plots

This study was conducted in three permanent forest plots situated in the Changbai Mountain Nature Reserve, which is located in Jilin Province of northeastern China (Figure 1). The region is characterized with a typical continental mountain climate which is affected by the monsoon seasons. The mean annual temperature at the site was 3.6°C, with an average monthly temperature ranging from a minimum of −15.4°C in January to a maximum of 19.6°C in August. The mean annual precipitation in the study was determined to be 707 mm, and the mean relative humidity was approximately 72%. The primary vegetation type was observed to be a broad‐leaved Korean pine (*Pinus koraiensis*) mixed forest. The dominant species include *Pinus koraiensis*, *Tilia amurensis*, *Abies nephrolepis*, and *Acer pseudo‐sieboldianum*.

From 2005 to 2007, three equally sized permanent plots were established as follows: A secondary conifer and broad‐leaved mixed forest (CBF); a secondary poplar and birch mixed forest (PBF); and a mixed Tilia and Korean pine forest (TKF), respectively, as detailed in Table 1. Each of the plots covered 5.2 hectares (260 × 200 m) and was divided into 130 quadrats of 20 × 20 m. All individuals with a diameter at the breast (DBH) ≥ 1 cm within the plots were identified, tagged, mapped, and measured. In this study's analyses, only individuals with DBH ≥ 5 cm were included, since such trees were determined to account for almost all of the above‐ground biomass (AGB) and productivity (Chiang et al., 2016). In addition, all of the aforementioned plots were re‐censused 5 years after being first established.

**TABLE 1:** Description of the three permanent plots

	CBF	PBF	TKF
Land‐use History	Large‐sized trees were cut during the Japanese War in 1931–1945	Clear‐felled in the 1930s	Primary forest which has never been cut
Forest type	Secondary conifer and broad‐leaved forest	Secondary poplar and birch mixed forest	Mixed Tilia and Korean pine forest
Location	42°20.907′N 128°07.988′E	42°19.1667′N 128°07.817′E	42°13.684′N 128°04.573′E
Altitude (m)	813 (798–826)	879 (865–894)	1,021 (1005–1034)
Dominant species (based on basal area)	*Tilia amurensis*, *Betula platyphylla*, *Abies nephrolepis*	*Betula platyphylla*, *Tilia amurensis*, *Populus davidiana*	*Pinus koraiensis*, *Tilia amurensis*, *Populus cathayana*
Species composition	6,618 living trees, comprising 39 species and 22 genera	6,288 living trees, comprising 45 species and 23 genera.	4,667 living trees, comprising 22 species and 13 genera

### 2.2 Above‐ground biomass and productivity values

**FIGURE 2:**
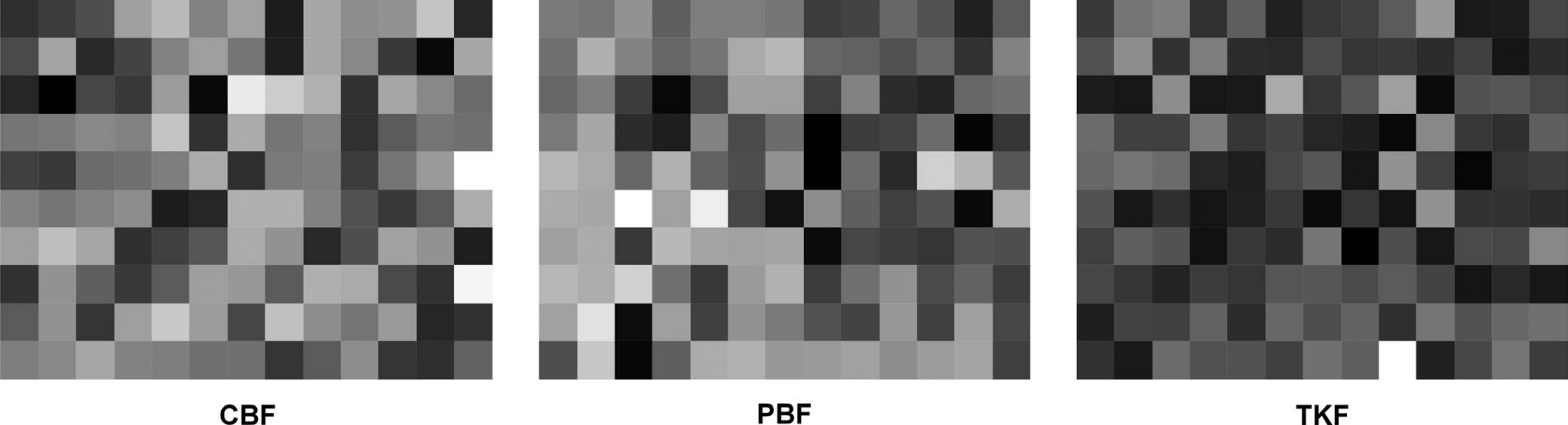
Initial above‐ground biomass AGB_i_ [ton ha^−1^ year^−1^]) patterns at the quadrat of 20 × 20 m in the three established plots. In the figure, the shading from light to dark indicates the observed values from low to high, respectively

All of the living individuals with DBH ≥ 5 cm during the first census were used to calculate the AGB. The AGB was determined using the regional‐specific allometric equations provided by He et al. (2018). The equation used in this study was as follows:

AGB=exp(a+b×ln(DBH))

where *a* and *b* represent the estimated coefficients. In the present study, 24.4% of species in the experimental plots own their specific allometric equations. For species with unknown coefficients, the coefficients of the most similar genus species were chosen. The AGB of each 20 × 20 m quadrat was calculated as the sum of the individual AGB values. Then, the initial above‐ground biomass (AGB_i_) for each quadrat was determined using the biomass data of the first census, scaled to the one hectare, as shown in Figure 2. In the analyses, we followed the accounting method for annual AGB increment (productivity) of Finegan et al. (2015) as follows: For each surviving individual, we calculate the annual AGB increment from the first census to the second census. Annual AGB increment of surviving trees (ΔAGB_sur_) was computed as the sum of annual AGB increment of all surviving trees at the quadrat level and then scaled to the hectare level. For each recruit which reached 5 cm DBH at the second census, annual AGB increment was estimated as the AGB minus the AGB of 5 cm DBH. Annual AGB increment of recruits (ΔAGB_rec_) was the sum of annual AGB increment of the recruits at the quadrat level and then scaled to the hectare level. Total annual AGB increment (ΔAGB_tot_) was the sum of ΔAGB_sur_ and ΔAGB_rec._

### 2.3 Biotic and abiotic variables

The biotic and abiotic variables were used to model the annual AGB increments (Table 2). In the present study, the species diversity, stand structural diversity, and density variables were used to represent the biotic variables. The species diversity included species richness (SR), rarefied species richness (Srare), and phylogenetic diversity (PD). The rarefied species richness considered the tree density effects and represented the number of species observed when a certain number of trees were randomly drawn from a quadrat (Poorter et al., 2015). In addition, this study calculated PD based on a phylogenetic tree, following the research approach of Qian and Jin (2016). First, the standardized nomenclature of the 49 species encountered in the three experimental plots was checked according to The Plant List (www.theplantlist.org), which is a popular international standard database for plant nomenclature (Hao, Ganeshaiah, Zhang, Zhao, & von Gadow, 2019). Then, the “S.PhyloMaker” package in R was used to generate a phylogenetic tree (Qian & Jin, 2016). The PD for each quadrat was calculated as the sum of branch lengths (Faith, 1992). The structural diversity included the coefficient of variation of the DBH of the trees in a stand (CV) which represented the size inequalities of individual specimens and the maximum DBH (MAX). The forest density included the number of stems (Nstems) and the initial above‐ground biomass (AGB_i_) of each quadrat.

**TABLE 2:** Descriptive statistical data for the response and predictor variables used to test the effects of the biotic and abiotic attributes on the productivity levels in the three established plots

	CBF			PBF			TKF		
	Mean ± *SD*	Max	Min	Mean ± *SD*	Max	Min	Mean ± *SD*	Max	Min
Annual AGB increment of surviving trees (ΔAGB_sur_ [ton ha^−1^ year^−1^])	1.92 ± 0.85	5.38	0.34	2.68 ± 0.74	4.85	1.00	1.59 ± 0.92	4.56	0.00
Annual AGB increment of recruit trees (ΔABG_rec_ [ton ha^−1^ year^−1^])	0.02 ± 0.03	0.18	0.00	0.04 ± 0.05	0.31	0.00	0.02 ± 0.02	0.10	0.00
Total annual AGB increment (ΔAGB_tot_ [ton ha^−1^ year^−1^])	1.94 ± 0.85	5.38	0.37	2.72 ± 0.74	4.87	1.04	1.6 ± 0.93	4.58	0.00
Biotic									
Species Richness (SR)	11.53 ± 2.5	17.00	5.00	11.35 ± 2.26	18.00	6.00	6.32 ± 1.47	11.00	3.00
Rarefied species diversity (Srare)	7.9 ± 1.29	11.00	3.79	7.91 ± 1.24	10.61	4.62	5.37 ± 1.04	8.00	3.00
Phylogenetic diversity (PD)	1604.17 ± 292.35	2,177.30	705.72	1595.17 ± 273.87	2,240.12	751.16	1,180.76 ± 130.51	1,580.64	855.32
Coefficient variation of DBH (CV)	0.75 ± 0.13	1.01	0.49	0.53 ± 0.11	1.05	0.33	0.85 ± 0.14	1.36	0.52
Maximum DBH (MAX [m])	53.38 ± 11.53	85.00	29.80	36.25 ± 10.27	105.00	24.00	68.49 ± 12.27	147.70	49.90
Number of stems (Nstems)	50.9 ± 13.96	84.00	20.00	48.28 ± 13.12	94.00	23.00	35.9 ± 7.48	65.00	18.00
Initial AGB (AGB_i_ [ton ha^−1^ year^−1^])	114.24 ± 31.36	221.32	34.53	75.58 ± 20.2	142.66	27.21	206.38 ± 55.65	525.23	70.35
Abiotic									
Elevation (ELE [m])	813.06 ± 2.05	816.62	808.02	878.51 ± 2.11	884.99	875.32	1,020.56 ± 1.97	1,024.53	1,015.85
Aspect (ASP)	−0.03 ± 0.46	1.00	−0.99	−0.54 ± 0.6	1.00	−1.00	0.1 ± 0.63	1.00	−1.00
Slope (SLO)	3.87 ± 3.63	12.60	0.59	4.05 ± 2.17	9.98	0.51	2.52 ± 1.61	12.08	0.45
Convexity (CON)	−0.01 ± 1.34	3.20	−2.83	0.05 ± 1.99	5.21	−3.22	0.01 ± 0.61	1.40	−2.20
Soil depth (DEP [cm])	35.95 ± 12.72	90.00	14.00	46.57 ± 15.28	129.00	12.00	27.92 ± 7.44	50.00	10.00
Soil total nitrogen (N)	0.49 ± 0.29	1.53	0.04	0.31 ± 0.13	0.66	0.01	0.37 ± 0.15	1.01	0.04
Soil total phosphorus (P)	0.01 ± 0	0.01	0.00	0.01 ± 0	0.02	0.00	0.06 ± 0.02	0.10	0.02
Soil total potassium (K)	2.28 ± 0.62	4.90	0.80	2.16 ± 0.63	4.20	0.80	2.36 ± 0.7	3.94	0.20
Organic matter (OM)	17.22 ± 8.65	44.29	2.66	16.79 ± 9.22	41.88	1.47	16.46 ± 9.17	41.10	0.49
Soil water content (SW)	54.79 ± 10.99	86.93	30.08	30.95 ± 11.4	74.93	15.85	28.19 ± 3.46	34.53	13.18

Note

Mean ± *SD* refers to the average and standard deviations; Max indicates the maximum value; Min refers to the minimum value; and AGB represents above‐ground biomass

For the abiotic variables, this study measured four topographic variables, including elevation (ELE), aspect (ASP), slope (SLO), and convexity (CON), and six edaphic variables including soil depth (DEP), soil total nitrogen (N), soil total phosphorus (P), soil total potassium (K), organic matter (OM), and soil water content (SW). The ELE of a particular 20 × 20 m quadrat was estimated as the mean elevation of its four vertexes. Then, the ASP, SLO, and CON of each quadrat were calculated utilizing the elevation value. In each 20 × 20 m quadrat, two soil samples were collected from the top 20 cm layer randomly. The mean value of the two samples for four soil variables, including N, P, K, and OM, was measured for each quadrat (Tan, Fan, Zhang, von Gadow, & Fan, 2017). DEP and SW were measured in each quadrat. In particular, SW was measured using a soil moisture meter (Delta‐T).

### 2.4 Data analysis

Prior to completely the analysis of the acquired data, the correlations between the annual AGB increments and the explanatory variables were first tested using a Pearson correlation method (Table S1). Then, the variance inflation factor (VIF) was used to test the multicollinearity using the R package “car” (Fox & Monette, 1992). The explanatory variables were selected by comparing VIF results. A VIF > 10 indicated excessive collinearity (Marini et al., 2011; Myers, 1990). The SR and ELE were removed due to the high VIF (VIF > 10), and the final model included two species diversity variables (Srare and PD); two structure diversity variables (CV and MAX); two density variables (Nstems and AGBi); three topographic variables (ASP, SLO, and CON); and six edaphic variables (DEP, N, P, K, OM, and SW).

Linear models were first constructed in this study for the purpose of testing the singular effects of each explanatory variable on the annual AGB increments (ΔAGB_sur_, ΔAGB_rec_, and ΔAGB_tot_). Then, a series of linear mixed‐effects models (LMM) were used to estimate how the different biotic or abiotic predictors influenced the annual AGB increments (ΔAGB_sur_, ΔAGB_rec_, and ΔAGB_tot_). Prior to the analyses, all of the responses and explanatory variables were scaled. In this study's models, all of the biotic and abiotic variables (with the exceptions of the SR and ELE) were used as the fixed effects. Meanwhile, the forest plot was treated as a random effect. In order to account for the spatial autocorrelations in the contiguous quadrats, a distance‐based spherical variogram was used in the LMM models to describe the spatial structure. The spherical variogram model is commonly used in the geostatistical analysis of ecological data, because its structure of linear increase at the origin, followed by stabilization to an asymptote, corresponds with the spatial variation that is often observed in nature (Fleishman & Mac Nally, 2006; Kleisner, Walter, Diamond, & Die, 2010; Lloyd, 2010). In order to determine the confidence intervals of the model coefficients, a bootstrapping approach was employed. This study randomly selected 300 quadrats from all the 390 quadrats as one training set for the purpose of calculating the coefficients of the biotic and abiotic predictors each time. Then, the process was repeated 1,000 times in order to determine the means and standard errors of the model coefficients (Efron & Tibshirani, 1993).

The relative importance of the different processes on the annual AGB increments was tested by comparing the following ten models: (a) Two null models without fixed effects (Null Model 2 considered the spatial autocorrelation); (b) Four biotic models containing species diversity (Srare, PD), structural diversity (CV and MAX), and density (Nstems and AGB_i_) variables as fixed effects; (c) Three abiotic models containing topographic (ASP, SLO, and CON) and edaphic (DEP, N, P, K, OM, and SW) variables as fixed effects; and (d) A fully saturated model in which all of the biotic and abiotic variables were contained as fixed effects. All of the aforementioned models included plot as a random effect. Then, the results of the ten groups of models were compared using the Akaike information criterion (AIC). All of the analyses were implemented using the “nlme” package (Pinheiro et al., 2016) in R 3.5.1 (R Core Team, 2019).

## 3 RESULTS

**FIGURE 3:**
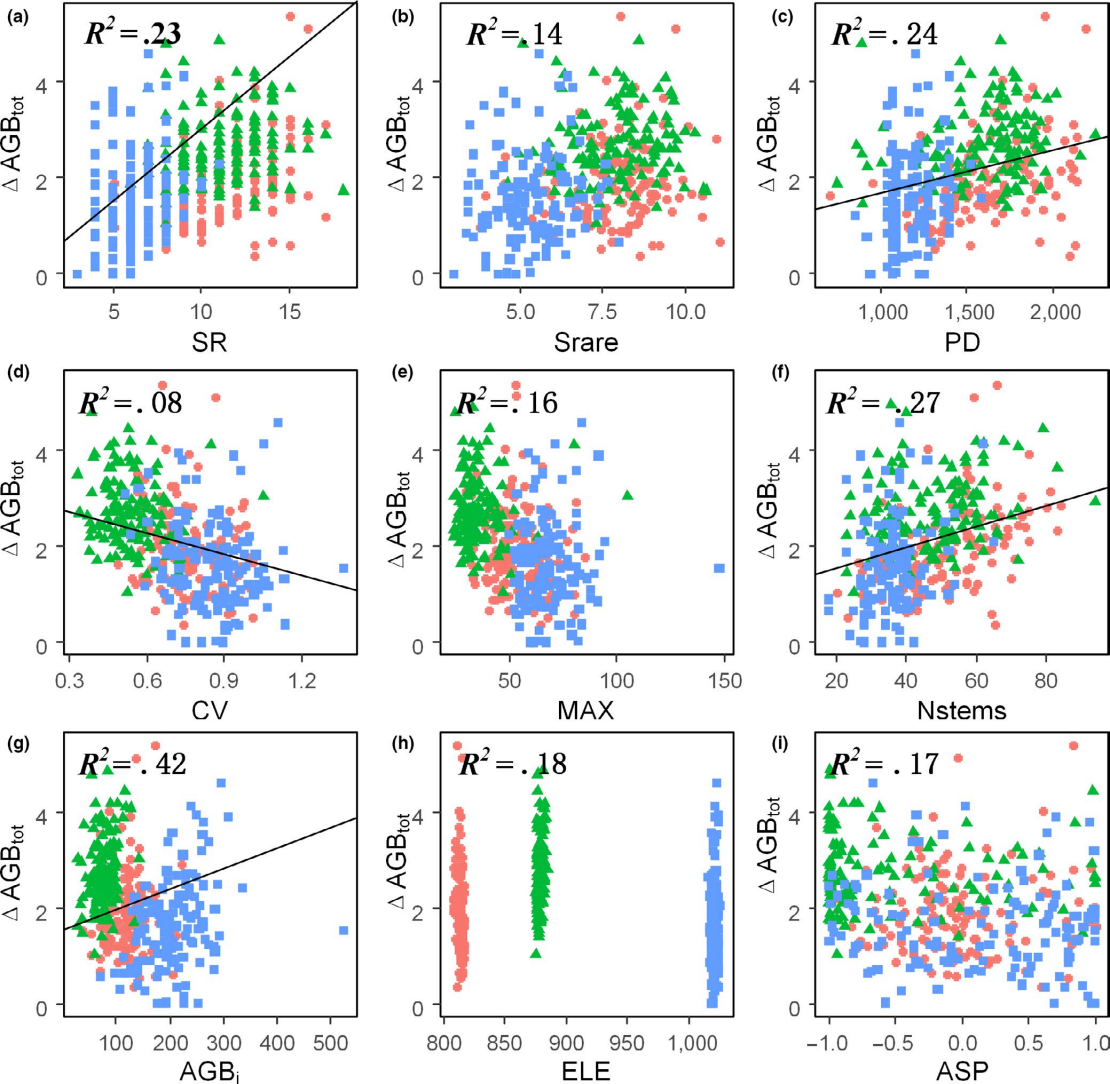
Bivariate relationships between: (a) Species richness; (b) Rarefied species richness; (c) Phylogenetic diversity; (d) Coefficient of variation of diameter at the breast; (e) Maximum diameter at breast height; (f) Number of stems; (g) Initial above‐ground biomass; (h) Elevation; (i) Aspect and total annual above‐ground biomass increment (ΔAGB_tot_ [ton ha^−1^ year^−1^]). ΔAGB_tot_ in the CBF is indicated by red circles; PBF by green triangles; and the TKF by blue squares. Black lines represent statistically significant effects (*p* < .05), and the figures without lines indicate nonsignificant effects (*p* > .05)

A series of bivariate models were adopted to test the biotic or abiotic effects on the annual AGB increments. According to the results, it was found that the ΔAGB_sur_ had displayed significant positive relationships with the SR, PD, Nstems, and AGB_i_, but negative relationships with the CV, OM, and SW (Figures S1 and S2). In regard to the ΔAGB_rec_, it was found to be positively related to the Srare, CV, N, and SW, while negatively related to the Nstems, AGB_i_, ASP, and K (Figures S3 and S4). In addition, it was observed that the ΔAGB_tot_ (e.g., ΔAGB_tot_ = ΔAGB_sur_ + ΔAGB_rec_) was mainly determined by the ΔAGB_sur_. Therefore, it was also significantly positively related to the SR, PD, Nstems, and AGB_i_, but negatively related to the CV, OM, and SW (Figures 3 and S5). It was observed that among the biotic variables, the AGB_i_ explained the greatest percentage of the variations in the ΔAGB_sur_ and ΔAGB_tot_, and the CV explained the greatest percentage of the variations in the ΔAGB_rec_.

**FIGURE 4:**
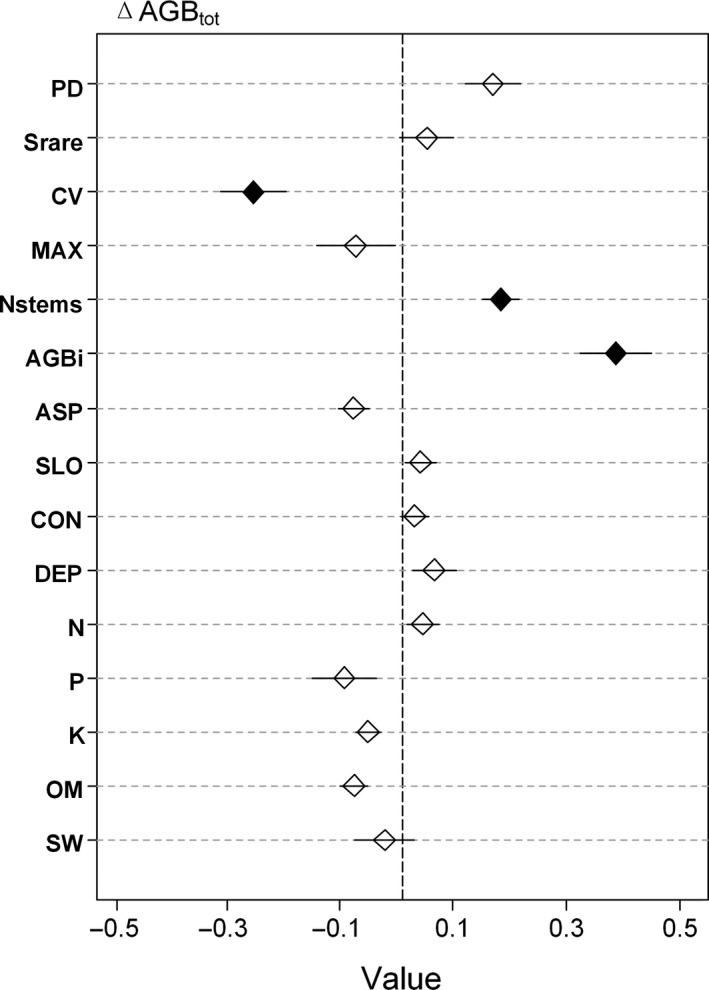
Linear mixed model results of productivity: ΔAGB_tot_, annual above‐ground increment of the total community [ton ha^−1^ year^−1^]) using rarefied species diversity (Srare), phylogenetic diversity (PD), coefficient of variation of diameter at the breast (CV), maximum of diameter at the breast (MAX), number of stems (Nstems), initial forest biomass (AGB_i_), aspect (ASP), slope (SLO), and convexity (CON), soil depth (DEP), soil total nitrogen (N), soil total phosphorus (P), soil total potassium (K), organic matter (OM), and soil water content (SW). Each variable was standardized, and their effect sizes were compared in order to determine the differences in those indicators. Closed rhombus indicates a significant effect on productivity (*p* < .05), and the lines indicate the standard errors

**FIGURE 5:**
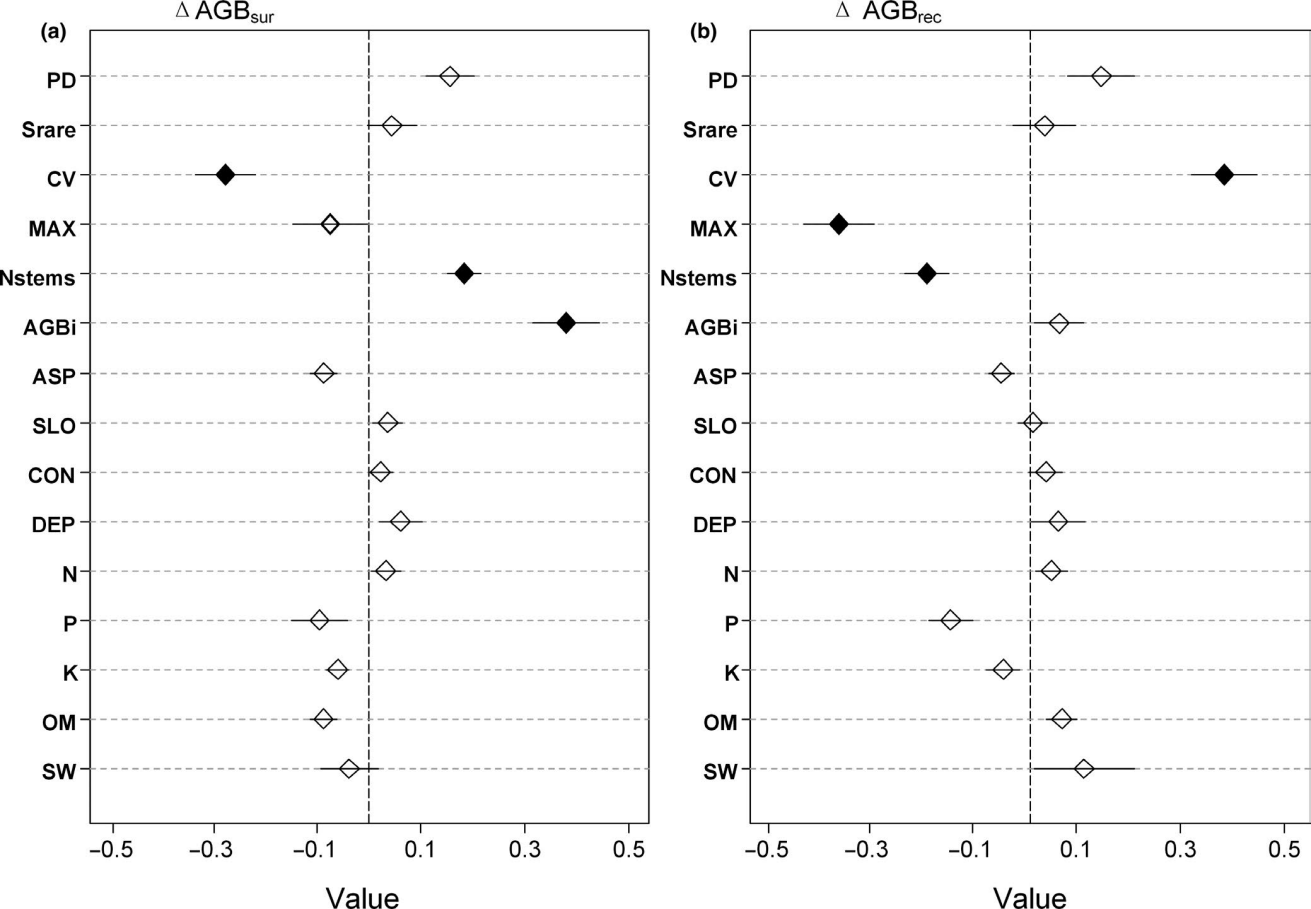
Linear mixed model results of productivity: (a) ΔAGB_sur_, annual above‐ground biomass increment of surviving trees [ton ha^−1^ year^−1^]; (b) ΔAGB_rec_, annual above‐ground biomass increment of recruit trees [ton ha^−1^ year^−1^] using rarefied species diversity (Srare), phylogenetic diversity (PD), coefficient of variation of diameter at the breast (CV), maximum of diameter at the breast (MAX), number of stems (Nstems), initial forest biomass (AGB_i_), aspect (ASP), slope (SLO), and convexity (CON), soil depth (DEP), soil total nitrogen (N), soil total phosphorus (P), soil total potassium (K), organic matter (OM), and soil water content (SW). Each variable was standardized, and their effect sizes were compared in order to determine the differences in those indicators. Closed rhombus indicates a significant effect on productivity (*p* < .05), and the lines indicate the standard errors

To further detach the relative importance of each variable, we conducted 1,000 times replacement and got the coefficients and standard errors of each variable. The results revealed that the AGB_i_ was the most important indicator of the ΔAGB_sur_ and ΔAGB_tot_. Meanwhile, the CV had the next strongest effects, followed by the Nstems, as detailed in Figures 4 and 5. It was observed that the AGB_i_ and Nstems were positively related to the ΔAGB_sur_ and ΔAGB_tot_, and the CV was found to have negative effects on the ΔAGB_sur_ and ΔAGB_tot_. In regard to the ΔAGB_rec_, among the structural variables, the CV had strong positive effects, while the MAX had strong negative effects, and the Nstems displayed negative effects. In addition, it was found that all of the species diversity and abiotic variables had nonsignificant effects on the annual AGB increments.

The next step in this research study was to determine whether or not the annual AGB increments were driven by biotic or abiotic divers. Subsequently, ten alternative models were used to compare the AIC criteria. The biotic models were proven the best models for the ΔAGB_sur_, ΔAGB_rec_, and ΔAGB_tot_ (Table 3)_._ Among the models, three biotic models (species diversity, structural diversity, and density models) and the density models were found to be the most accurate models for the ΔAGB_sur_ and ΔAGB_tot_, and the ΔAGB_rec_ was best explained by this study's structural diversity model.

**TABLE 3:** Comparison of the results of the linear mixed‐effect models in predicting the productivity levels of the three temperate forests

	ΔAGB_sur_					ΔAGB_rec_					ΔAGB_tot_			
	ΔAIC	Loglik	*R* ^2^m	*R* ^2^c	ΔAIC		Loglik	*R* ^2^m	*R* ^2^c	ΔAIC		Loglik	*R* ^2^m	*R* ^2^c
Null														
Null model 1	79.90	−508.89	0.00	0.22	29.53		−542.17	0.00	0.07	77.96		−507.14	0.00	0.23
Null model 2	44.65	−489.27	0.00	0.19	23.45		−537.13	0.00	0.07	42.26		−487.29	0.00	0.19
Biotic														
Species diversity model	44.34	−487.11	0.08	0.25	14.51		−530.66	0.06	0.08	41.79		−485.06	0.08	0.25
Structural diversity model	28.57	−479.23	0.09	0.09	8.94		−527.87	0.05	0.14	28.92		−478.62	0.07	0.14
Density model	23.55	−476.72	0.10	0.40	16.76		−531.78	0.04	0.06	10.95		−469.64	0.07	0.33
Abiotic														
Edaphic model	45.84	−483.86	0.04	0.04	17.51		−528.16	0.12	0.22	42.53		−481.43	0.05	0.12
Topographic model	49.70	−488.79	0.01	0.10	27.89		−536.35	0.00	0.06	47.23		−486.78	0.01	0.11
Grouped														
Biotic model	0.00	−460.94	0.19	0.32	0.00		−519.40	0.10	0.14	0.00		−460.16	0.19	0.32
Abiotic model	48.53	−482.21	0.06	0.11	21.68		−527.24	0.12	0.21	46.64		−480.48	0.06	0.12
Full														
Full model	10.31	−459.10	0.26	0.34	4.48		−514.64	0.18	0.25	11.27		−458.80	0.26	0.35

Note

**Δ**AIC refers to delta Akaike information criterion; Loglik is the log‐likelihood value; *R* ^2^m indicates the marginal R squared; *R* ^2^c is the conditional R squared; **Δ**AGB_sur_ refers to the annual above‐ground biomass increment due to the surviving trees (ton ha^−1^ year^−1^); ΔAGBG_rec_ indicates the annual AGB increment due to the recruit trees (ton ha^−1^ year^−1^); and ΔAGB_tot_ is the total annual AGB increment (ton ha^−1^ year^−1^) The null models included no fixed effects. However, Null Model 2 considered the spatial autocorrelations. The biotic models included the species diversity (rarefied species richness and phylogenetic diversity); structural diversity (coefficient of variation of diameter at the breast and the maximum of diameter at the breast); and density (number of stems and initial forest biomass) variables. The abiotic models included the soil (soil depth, soil total nitrogen, soil total phosphorus, soil total potassium, organic matter, and water content) and topographic (aspect, slope and convexity) variables. The full models included all of the biotic and abiotic variables.

## 4 DISCUSSION

The objective of this study was to assess the relative importance of the biotic and abiotic variables on the productivity levels in the three established temperate forest plots. It was found that the biotic models performed better than the abiotic models. Structural diversity had significantly negative effects on the annual AGB increments of the surviving trees (ΔAGB_sur_) and the total tree community (ΔAGB_tot_), while positive effects on the annual AGB increments of the recruit trees (ΔAGB_rec_). In addition, the density variables displayed positive effects on the ΔAGB_sur_ and ΔAGB_tot_, but negative effects on the ΔAGB_rec_. The species diversity was determined to have nonsignificant effects on all three components of productivity, including the ΔAGB_sur_, ΔAGB_rec_, and ΔAGB_tot_.

### 4.1 Nonsignificant species diversity effects on productivity

Despite the fact that the majority of the previous studies had observed positive species diversity effects on productivity at global and regional scales (Liang et al., 2016; Ratcliffe et al., 2016; Ruiz‐Benito et al., 2014), this study observed nonsignificant species diversity effects when compared with other biotic variables in the three forest plots. However, the results obtained in this study were in line with some of the previous studies conducted in temperate (Fotis et al., 2018), subtropical (Wu, Wang, Wu, Xia, & Fang, 2015), and tropical forests (van der Sande et al., 2018). It is known that niche partitioning or facilitation among species will contribute to ameliorating resource availability efficiency (niche complementary effect), which in turn will enhance forest productivity in communities with high species diversity (Tilman et al., 1997). However, the identities of the species may also shape the diversity and productivity relationships at the community level (selection effect) (Loreau & Hector, 2001). For example, Cheng, Zhang, Zhao, and von Gadow (2018) observed that the dominant species tended to determine the negative diversity effects on productivity within the same area. The directions (positive or negative) of the effects may be combinations of niche complementary and selection effects. Furthermore, nonsignificant effects may have been caused by the balance between the niche complementary effects and the selection effects. Additionally, nonsignificant effects may also have been caused by functional redundancy. In other words, adding to the species richness will not contribute to the niche complementarity (McCann, 2000; Peterson, Allen, & Holling, 1998). Moreover, other related studies have reported species diversity may mediate functional trait diversity, and subsequently alter forest productivity (Chiang et al., 2016; Hao et al., 2018). In the present study, the focus was placed on the diversity of the species. However, it is recommended that future studies should consider not only the diversity of species, but also the diversity of the functional traits of the species.

### 4.2 Structural diversity had mixed effects on productivity

The three components of productivity which were measured in this study did not respond in the same way to the effects of structural diversity. It was found that the structural diversity had positive effects on the increments of the recruit trees (ΔAGB_rec_), yet negative effects on the surviving trees (ΔAGB_sur_). Generally speaking, structural diversity reflects the tree size inequality and to some extent can reflect the complementary effects of the canopy. It has been observed that as tree size inequality increases, the availability of light and vertical spaces would be expected to also increase (Zhang & Chen, 2015). In the study area, consistent with the aforementioned expectations, the recruit trees had benefited from high structural diversity, which was consistent with previous research findings in both temperate (Zhang & Chen, 2015) and subtropical forests (Fotis et al., 2018; Ouyang et al., 2019). However, for the surviving trees, the negative effects of the structural diversity included increased asymmetric competition for light resources (Weiner & Thomas, 1986). Since the recruit trees were essentially small trees located in the understory where the light was limited, improvements in the light conditions tended to increase their growth rates. However, the surviving trees were mostly large‐sized trees in the overstorey which had escaped from the competition for light, and may be not benefited from diverse vertical structures (Ali, 2019; Ali et al., 2019b). Additionally, the successional stage may offer a proper explanation for the negative structural diversity effects. The biotic variables and productivity change across succession. Compared to the old‐growth forests (TKF), two successional plots (CBF and PBF) have higher productivity and lower DBH variability. The negative relationship may be due to the higher productivity with lower CV values. The negative relationships observed in the present study were found to be consistent with previous studies in monocultures (Bourdier et al., 2016; Cordonnier & Kunstler, 2015), although the majority of such studies conducted in mixed forest environments had detected positive (Fotis et al., 2018; Ouyang et al., 2019; Zhang & Chen, 2015) or nonsignificant structural diversity effects (Yuan et al., 2018). Such mixed structural diversity effects have seldom been mentioned in previous research. Therefore, future studies should consider tree demographics, along with greater numbers of forest structural variables.

### 4.3 Density variables as the major drivers of productivity

The density variables were the most important drivers of the forest productivity in this study, showing positive impacts on the increments of the surviving trees. It was found that the obtained results were in line with other previous research findings in tropical (Lohbeck et al., 2015; van der Sande et al., 2017), subtropical forests (Chiang et al., 2016; Ouyang et al., 2019), and temperate forests (Yuan et al., 2018, 2019). Furthermore, the results supported the vegetation quantity hypothesis, in which communities with larger biomass stock and crown areas may transform more energy (Lohbeck et al., 2015). However, the high stand density was observed to have reduced the growth rates of the recruit trees. One possible explanation was the competition for light resources had limited the growth rates of the recruit trees (Muscarella, Messier, Condit, Hubbell, & Svenning, 2018). Specifically, the majority of the recruit trees were at a disadvantage in the competition for resources. Therefore, overstorey (adult) trees may have negative impacts on recruit trees since the competition for shared resources may increase as density increases. It was believed that other processes which can be used to evaluate individual increments in tree neighborhoods may offer similar explanations. For example, Chen et al. (2016) found that focal trees grew more slowly under dense neighborhood conditions. Those results were in agreement with previous studies in which it had been observed that the negative density dependence of recruit trees was a stronger factor in biomass production than that of surviving trees (van der Sande et al., 2017; Yuan et al., 2019). Nonetheless, this study's results provided additional evidence regarding the importance of density variables in maintaining forest productivity and should be considered in future policy‐making and forest management processes. In particular, the small‐sized trees draw benefit from decreasing stand density; however, enhanced density ensures a certain level of stand productivity. Thus, a balance between competition reduction and high productivity should be considered by the forest managers. These results are particularly relevant in providing timely support for the recently enforced Chinese nationwide harvesting ban of natural forests (Dai et al., 2018; Liu et al., 2018).

Similar to structural diversity, stand density can also reflect the available resources and space occupation of communities and may also reflect intraspecific interactions to some extent (Fortunel et al., 2018). This study found that structural diversity and stand density had displayed converse effects on the annual AGB increments. For example, dense and homogeneous stand structure will contribute to the productivity of surviving trees. However, the recruit trees may have benefited from the openness and increased tree size inequality of the canopy areas. It should be pointed out that neither the structural variables nor density variables could accurately describe the competitiveness factors between the individual trees. In other words, the availability of light or space may result in a reduction of competition for the small‐sized trees, while not necessarily increasing the competition of the large‐ or medium‐sized trees (Kunz et al., 2019).

### 4.4 Effects of the abiotic variables

Abiotic variables, such as topographic variables and edaphic conditions, may influence resource availability and subsequently impact forest productivity levels (Ouyang et al., 2019; van der Sande et al., 2018). However, it was observed in this study that there were no overall significant effects of the abiotic variables in the established forested plots. It was believed that those results may have been caused by the rather similar environmental conditions between plots, such as the flat topography and homogeneous soil nutrition levels (Ni, Baiketuerhan, Zhang, Zhao, & von Gadow, 2014; Zhang, Zhao, Gao, & von Gadow, 2010). At the local scale, the contributions of the abiotic variables may be related to variations in the biotic variables, such as habitat heterogeneity. Therefore, it was assumed that the abiotic variables would be more important drivers on the productivity under heterogeneous environmental conditions. The results achieved in this study may imply that significant effects of the abiotic variables could potentially be found at a regional scale level, or at a local scale when the habitats are heterogeneous.

## 5 CONCLUSIONS

The assessments of the relative importance of the biotic and abiotic drivers which determine forest productivity have been motivated by both a basic interest in understanding ecological mechanisms and the practical need to manage forest ecosystem functions and services. This study's findings highlighted the vital roles of biotic variables in determining productivity, yet abiotic variables did not contribute to tree productivity. Besides, it was found that density and structural diversity drivers played different roles in the growth rates of surviving trees and recruit trees, which may guide future temperate forest management processes. Therefore, this study emphasized the importance of simultaneously assessing the demographic processes of productivity when investigating the biotic and abiotic effects.

## CONFLICT OF INTEREST

The authors declare that they have no competing interests.

## AUTHOR CONTRIBUTIONS

**Qingmin Yue:** Methodology (lead); writing – original draft (lead). **Minhui Hao:** Methodology (equal); writing – original draft (equal). **Xiaoyu Li:** writing – review and editing (supporting). **Chunyu Zhang:** Project administration (lead); supervision (lead). **Klaus von Gadow:** writing – review and editing (supporting). **Xiuhai Zhao:** Project administration (lead); supervision (lead).

## Supporting information

Appendix S1

## Data Availability

The data that support the findings of this study can be accessed on Figshare: https://doi.org/10.6084/m9.figshare.12465242
