# Supplementary material for: Assessing biotic and abiotic effects on forest productivity in three temperate forests
Source: Ecol Evol. 2020 Jun 30;10(14):7887–900. doi: 10.1002/ece3.6516 (PMC7391343; doi:10.1002/ece3.6516)
Supplement: Supplementary file 1 — Appendix S1 [file ECE3-10-7887-s001.docx]

**Appendix S1**

**Table S1.** Correlations between response and predictor variables in the three temperate forests. ΔAGB_sur_: annual above-ground biomass increment of surviving trees [ton/ha/yr]; ΔAGB_rec_, annual above-ground biomass increment of recruit trees [ton/ha/yr]; ΔAGB_tot_, total annual above-ground increment [ton/ha/yr], species richness (SR), rarified species diversity (Srare), phylogenetic diversity (PD), number of stems (Nstems), initial forest biomass (AGBi), coefficient of variation of diameter at the breast (CV), the maximum of diameter at the breast (MAX), elevation (ELE), aspect (ASP), slope (SLO), convexity (CON), soil depth (DEP), soil total nitrogen (N),soil total phosphorus(P), soil total potassium(K), organic matter (OM), and soil water content (SW). The overstriking number means P value<0.05

|  | **ΔAGB_sur_** | **ΔAGB_rec_** | **ΔAGB_tot_** | **SR** | **Srare** | **PD** | **Nstems** | **AGBi** | **CV** | **MAX** | **ELE** | **ASP** | **SLO** | **CON** | **DEP** | **N** | **P** | **K** | **OM** | **SW** |
| --- | --- | --- | --- | --- | --- | --- | --- | --- | --- | --- | --- | --- | --- | --- | --- | --- | --- | --- | --- | --- |
| **ΔAGB_sur_** | **1** |  |  |  |  |  |  |  |  |  |  |  |  |  |  |  |  |  |  |  |
| **ΔAGB_rec_** | **0.18** | **1** |  |  |  |  |  |  |  |  |  |  |  |  |  |  |  |  |  |  |
| **ΔAGB_tot_** | **0.22** | **1** | **1** |  |  |  |  |  |  |  |  |  |  |  |  |  |  |  |  |  |
| **SR** | **0.18** | **0.4** | **0.4** | **1** |  |  |  |  |  |  |  |  |  |  |  |  |  |  |  |  |
| **Srare** | **0.27** | **0.3** | **0.31** | **0.92** | **1** |  |  |  |  |  |  |  |  |  |  |  |  |  |  |  |
| **PD** | **0.18** | **0.4** | **0.41** | **0.88** | **0.78** | **1** |  |  |  |  |  |  |  |  |  |  |  |  |  |  |
| **Nstems** | -0.05 | **0.38** | **0.38** | **0.56** | **0.29** | **0.59** | **1** |  |  |  |  |  |  |  |  |  |  |  |  |  |
| **AGBi** | **-0.26** | **-0.23** | **-0.24** | **-0.56** | **-0.58** | **-0.46** | **-0.24** | **1** |  |  |  |  |  |  |  |  |  |  |  |  |
| **CV** | **-0.1** | **-0.45** | **-0.45** | **-0.35** | **-0.31** | **-0.33** | **-0.28** | **0.63** | **1** |  |  |  |  |  |  |  |  |  |  |  |
| **MAX** | **-0.23** | **-0.38** | **-0.38** | **-0.44** | **-0.43** | **-0.36** | **-0.29** | **0.8** | **0.83** | **1** |  |  |  |  |  |  |  |  |  |  |
| **ELE** | **-0.15** | **-0.23** | **-0.24** | **-0.72** | **-0.67** | **-0.61** | **-0.48** | **0.67** | **0.37** | **0.49** | **1** |  |  |  |  |  |  |  |  |  |
| **ASP** | **-0.17** | **-0.24** | **-0.25** | **-0.19** | **-0.19** | **-0.16** | -0.06 | **0.35** | **0.35** | **0.37** | **0.16** | **1** |  |  |  |  |  |  |  |  |
| **SLO** | 0.04 | **0.12** | **0.12** | **0.24** | **0.22** | **0.24** | **0.17** | **-0.14** | -0.06 | -0.07 | **-0.23** | **0.2** | **1** |  |  |  |  |  |  |  |
| **CON** | 0.03 | 0.01 | 0.01 | 0.01 | 0.02 | -0.02 | -0.04 | -0.03 | 0.01 | -0.01 | 0.01 | -0.05 | 0 | 1 |  |  |  |  |  |  |
| **DEP** | **0.2** | **0.29** | **0.3** | **0.42** | **0.41** | **0.37** | **0.25** | **-0.45** | **-0.38** | **-0.39** | **-0.33** | **-0.19** | 0.01 | -0.03 | **1** |  |  |  |  |  |
| **N** | 0.03 | -0.07 | -0.07 | 0.08 | 0.1 | 0.1 | 0.03 | **0.01** | 0.11 | **0.1** | -0.17 | 0.08 | -0.02 | 0 | -0.03 | 1 |  |  |  |  |
| **P** | **-0.2** | **-0.32** | **-0.33** | **-0.69** | **-0.65** | **-0.58** | **-0.45** | **0.73** | **0.5** | **0.59** | **0.9** | **0.26** | **-0.24** | 0 | **-0.38** | -0.01 | 1 |  |  |  |
| **K** | **-0.14** | **-0.13** | **-0.13** | **-0.16** | **-0.22** | **-0.14** | 0.02 | **0.11** | 0.09 | 0.1 | 0.08 | 0.1 | 0.03 | 0.03 | **-0.11** | **-0.21** | 0.07 | 1 |  |  |
| **OM** | 0.07 | -0.07 | -0.07 | 0 | 0.03 | 0 | -0.04 | 0.01 | 0 | 0.04 | -0.03 | 0.03 | 0.06 | -0.07 | 0.09 | **0.36** | 0.04 | **-0.15** | 1 |  |
| **SW** | 0.1 | -0.05 | -0.04 | **0.43** | **0.41** | **0.37** | **0.23** | **-0.26** | **0.1** | -0.04 | **-0.64** | **0.12** | 0.08 | -0.04 | **0.18** | **0.31** | **-0.43** | -0.04 | 0.07 | 1 |

**
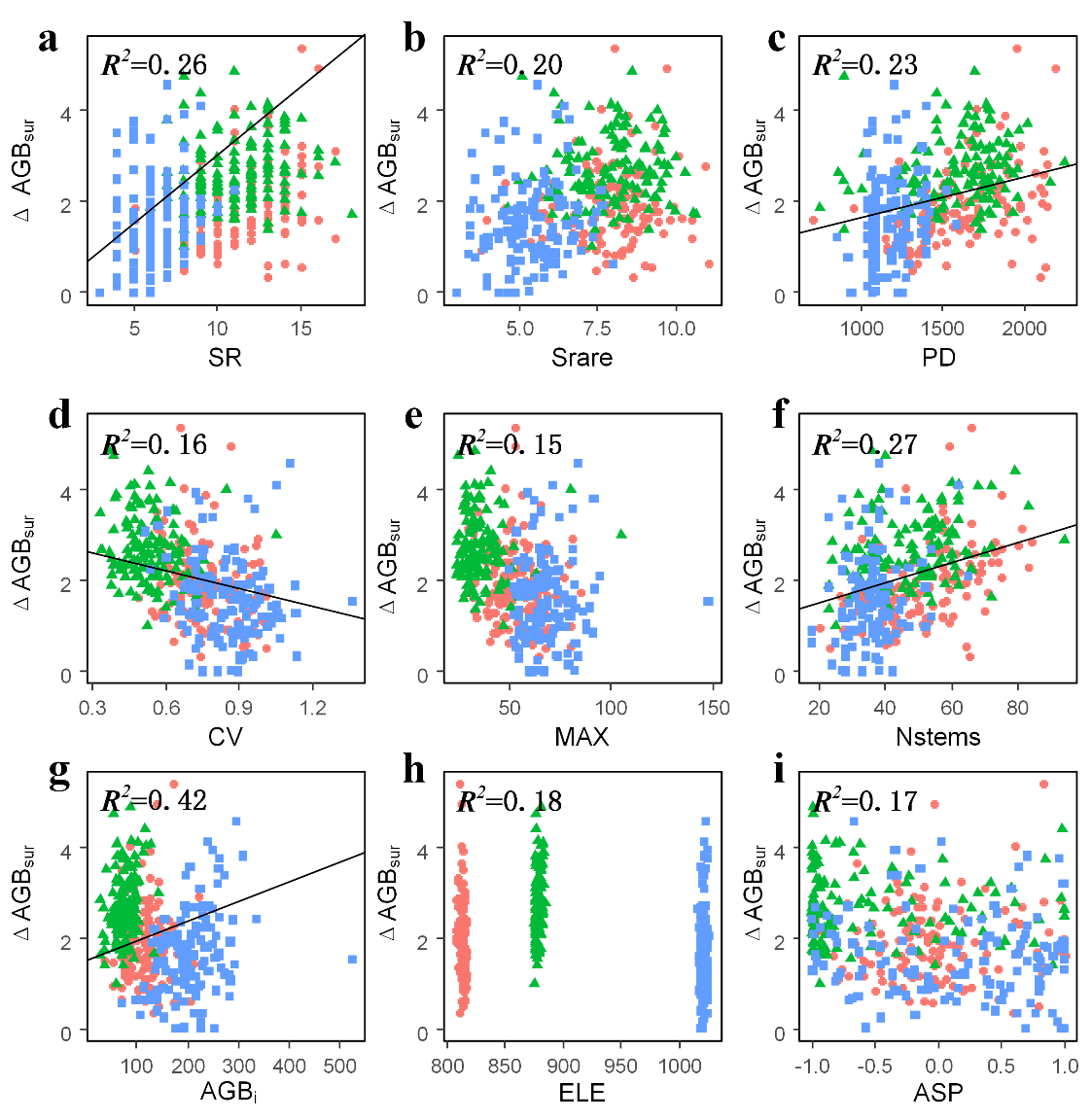
**

**Figure S1 Bivariate relationships between: (a) Species richness; (b) Rarified species richness; (c) Phylogenetic diversity; (d) Coefficient of variation of diameter at the breast; (e) Maximum diameter at breast height; (f) Number of stems; (g) Initial above-ground biomass; (h) Elevation; (i) Aspect and annual above-ground biomass increment of surviving trees (ΔAGB_sur_ [ton/ha/yr])****.** ΔAGB_sur_ in the CBF is indicated by red circles; PBF by green triangles; and the TKF by blue squares; Black lines represent statistically significant effects (P < 0.05), and the figures without lines indicate non-significant effects (P > 0.05) were observed


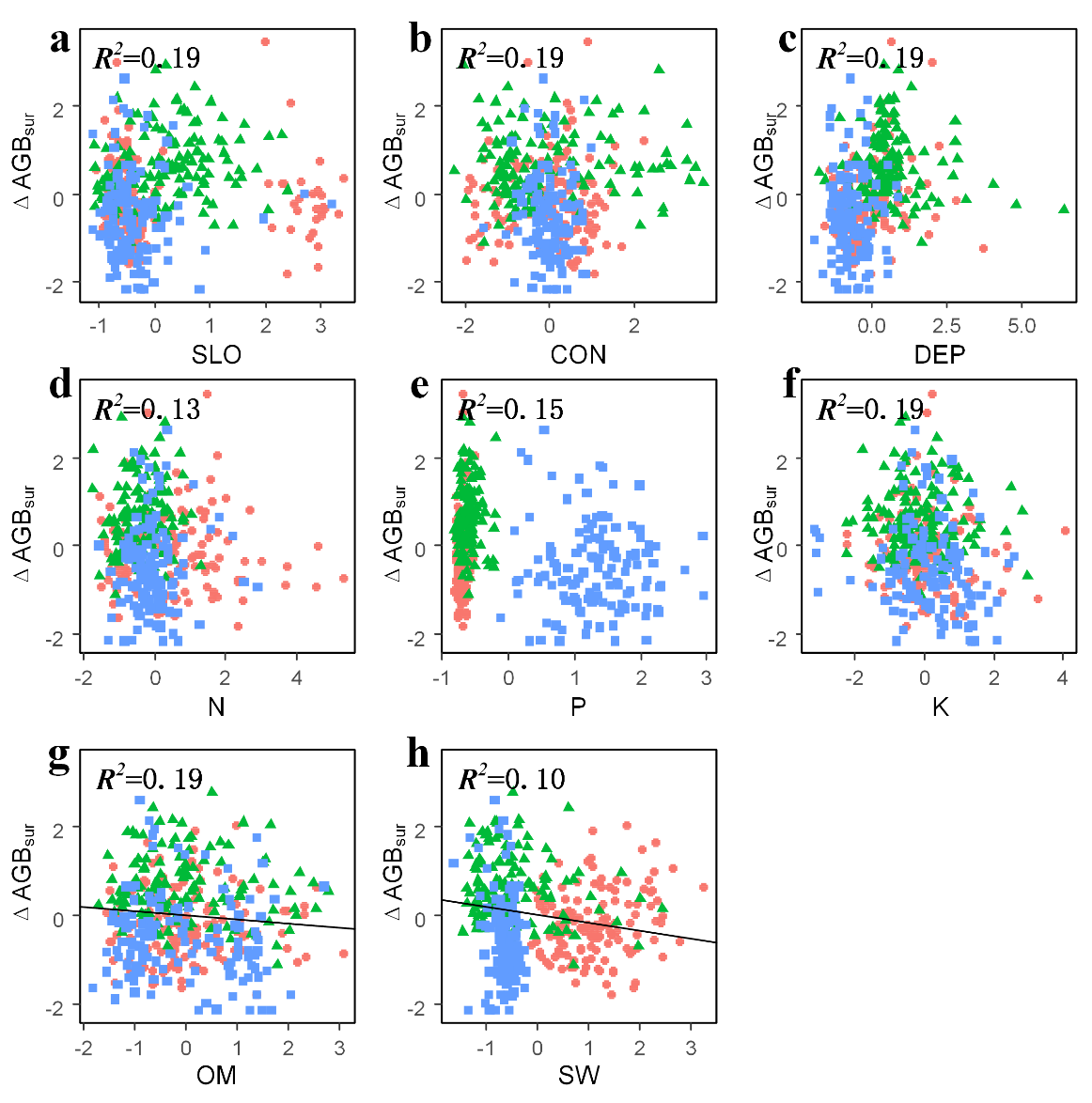


**Figure S2. Bivariate relationships between (a) Slope and (b) Convexity (c) Soil depth, (d) Soil total nitrogen, (e) Soil total phosphorus, (f) Soil total potassium, (g) Organic matter, (h) Soil water and annual above-ground biomass increment of surviving trees (ΔAGB_sur_ [ton/ha/yr]).** ΔAGB_sur_ in the CBF is indicated by red circles; PBF by green triangles; and the TKF by blue squares; Black lines represent statistically significant effects (P < 0.05), and the figures without lines indicate non-significant effects (P > 0.05) were observed

**
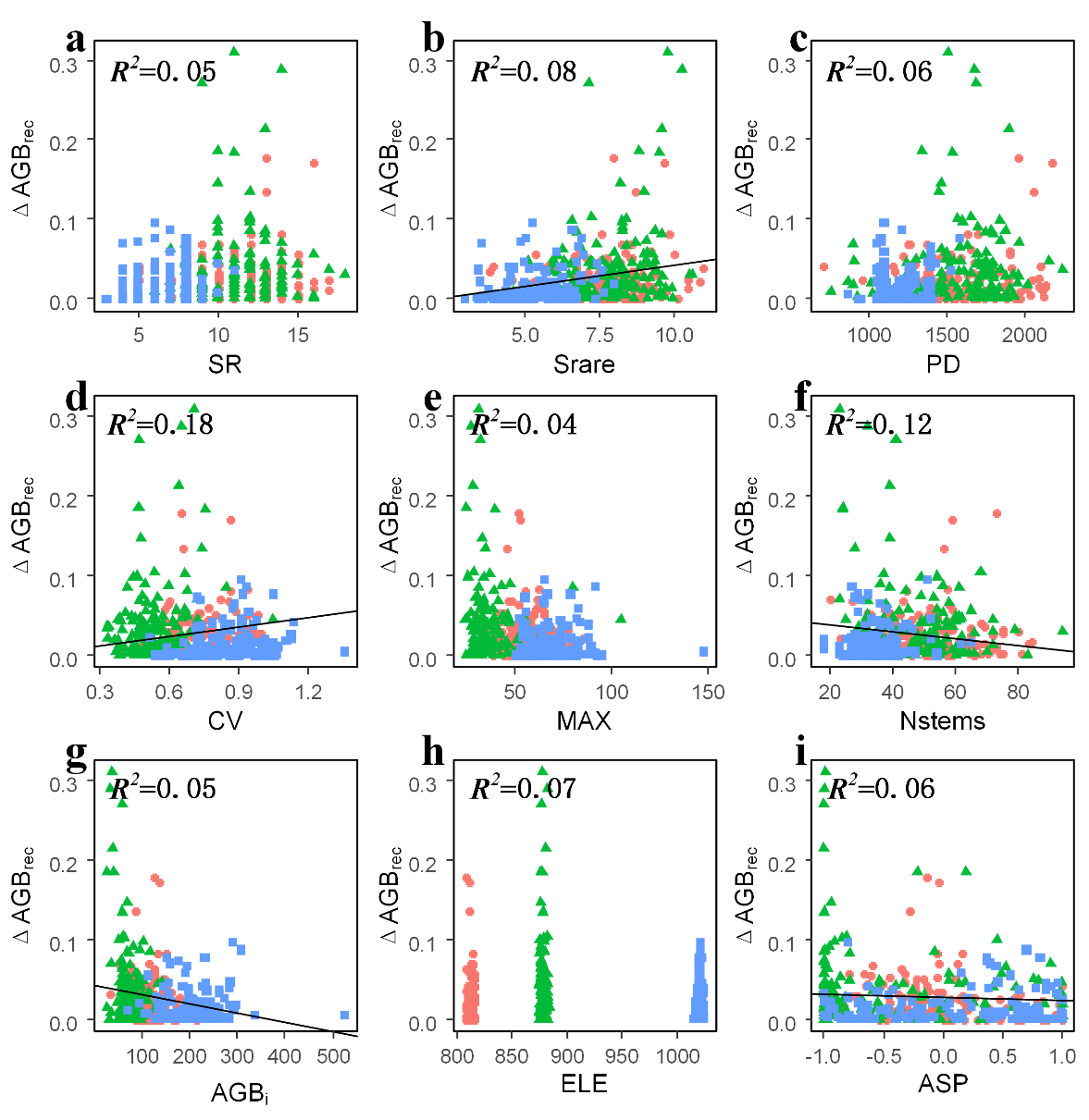
**

**Figure S3. Bivariate relationships between: (a) Species richness; (b) Rarified species richness; (c) Phylogenetic diversity; (d) Coefficient of variation of diameter at the breast; (e) Maximum diameter at breast height; (f) Number of stems; (g) Initial above-ground biomass; (h) Elevation; (i) Aspect and total annual above-ground biomass increment of recruit trees (ΔAGB_rec_ [ton/ha/yr]).** ΔAGB_rec_ in the CBF is indicated by red circles; PBF by green triangles; and the TKF by blue squares; Black lines represent statistically significant effects (P < 0.05), and the figures without lines indicate non-significant effects (P > 0.05) were observed


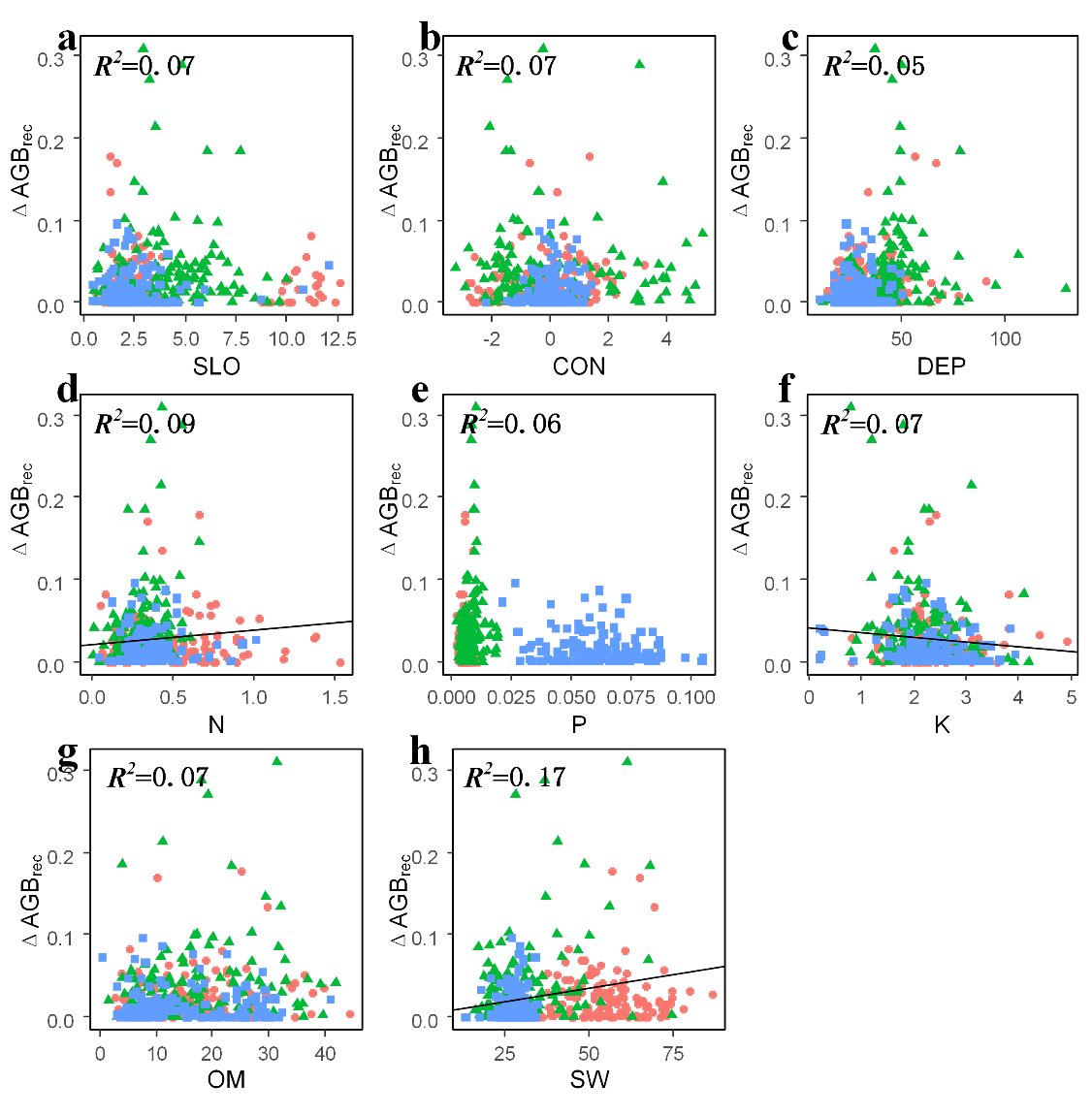


**Figure S4. Bivariate relationships between (a) slope and (b) convexity (c) soil depth, (d) soil total nitrogen, (e) soil total phosphorus, (f) soil total potassium, (g) organic matter, (h) soil water and total annual above-ground biomass increment of recruit trees (ΔAGB_rec_ [ton/ha/yr]).** ΔAGB_rec_ in the CBF is indicated by red circles; PBF by green triangles; and the TKF by blue squares; Black lines represent statistically significant effects (P < 0.05), and the figures without lines indicate non-significant effects (P > 0.05) were observed


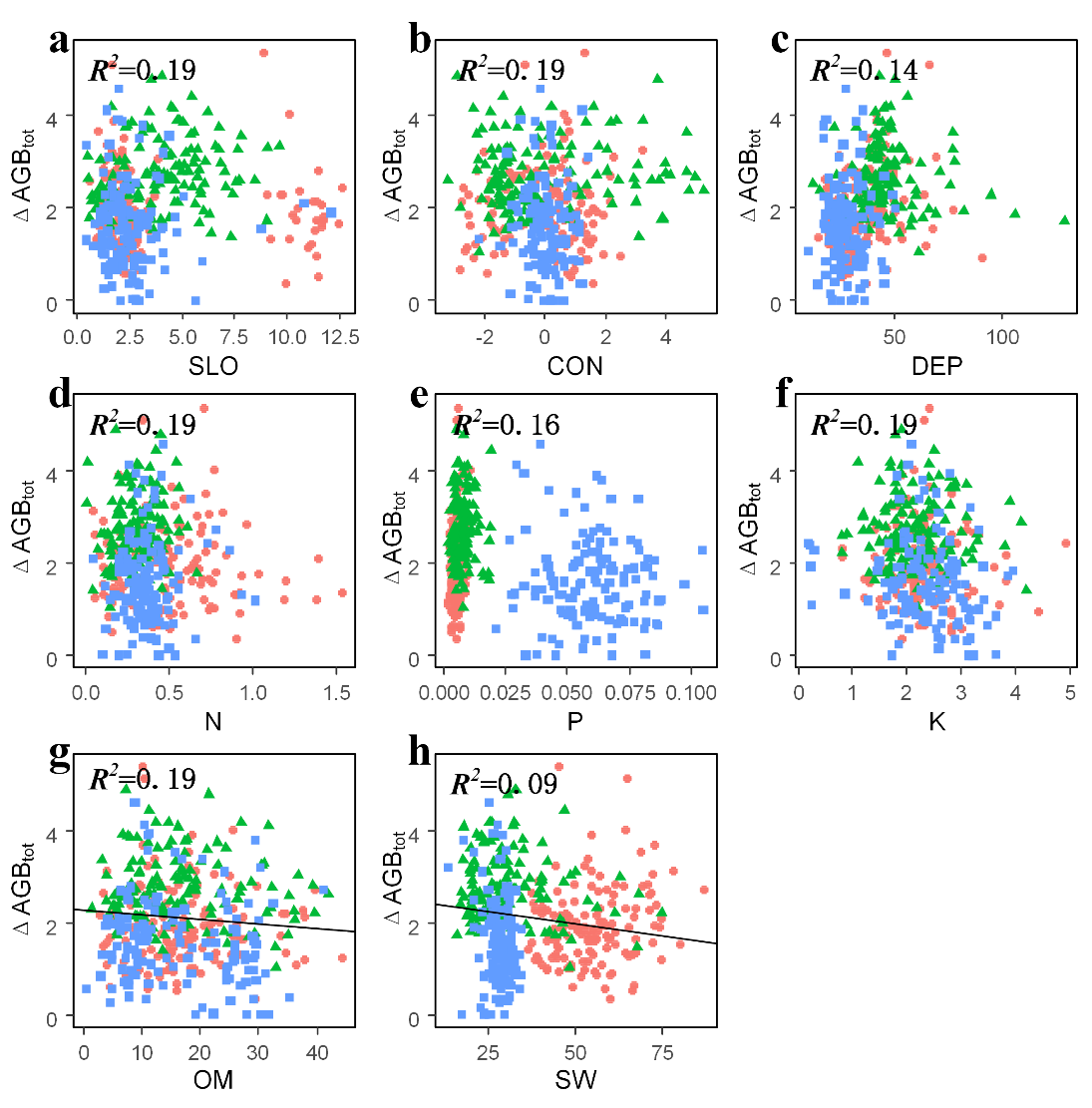


**Figure S5. Bivariate relationships between (a) slope and (b) convexity (c) soil depth, (d) soil total nitrogen, (e) soil total phosphorus, (f) soil total potassium, (g) organic matter, (h) soil water and total annual above-ground biomass increment (ΔAGB_tot_ [ton/ha/yr]).** ΔAGB_tot_ in the CBF is indicated by red circles; PBF by green triangles; and the TKF by blue squares; Black lines represent statistically significant effects (P < 0.05), and the figures without lines indicate non-significant effects (P > 0.05) were observed
